# Two-Dimensional Proton Magnetic Resonance Spectroscopy versus *J*-Editing for GABA Quantification in Human Brain: Insights from a GABA-Aminotransferase Inhibitor Study

**DOI:** 10.1038/s41598-018-31591-3

**Published:** 2018-09-04

**Authors:** Andrew P. Prescot, James J. Prisciandaro, Steven R. Miller, Gary Ingenito, Douglas G. Kondo, Perry F. Renshaw

**Affiliations:** 1 Department of Radiology and Imaging Sciences, University of Utah School of Medicine, Salt Lake City, UT USA; 2 Department of Psychiatry and Behavioral Sciences, Addiction Sciences Division, Medical University of South Carolina, Charleston, SC USA; 3 Catalyst Pharmaceuticals Inc, Coral Gables, FL USA; 4 Department of Psychiatry, University of Utah School of Medicine, Salt Lake City, UT USA; 5 Rocky Mountain Mental Illness Research, Education, and Clinical Center (MIRECC), Department of Veterans Affairs Medical Center, Salt Lake City, UT USA

## Abstract

Metabolite-specific, scalar spin-spin coupling constant (*J*)-editing ^1^H MRS methods have become gold-standard for measuring brain γ-amino butyric acid (GABA) levels in human brain. Localized, two-dimensional (2D) ^1^H MRS technology offers an attractive alternative as it significantly alleviates the problem of severe metabolite signal overlap associated with standard 1D MRS and retains spectroscopic information for all MRS-detectable species. However, for metabolites found at low concentration, a direct, *in vivo*, comprehensive methods comparison is challenging and has not been reported to date. Here, we document an assessment of comparability between 2D ^1^H MRS and *J*-editing methods for measuring GABA in human brain. This clinical study is unique in that it involved chronic administration a GABA-amino transferase (AT) inhibitor (CPP-115), which induces substantial increases in brain GABA concentration, with normalization after washout. We report a qualitative and quantitative comparison between these two measurement techniques. In general, GABA concentration changes detected using *J*-editing were closely mirrored by the 2D ^1^H MRS time courses. The data presented are particularly encouraging considering recent 2D ^1^H MRS methodological advances are continuing to improve temporal resolution and spatial coverage for achieving whole-brain, multi-metabolite mapping.

## Introduction

**Figure 1:**
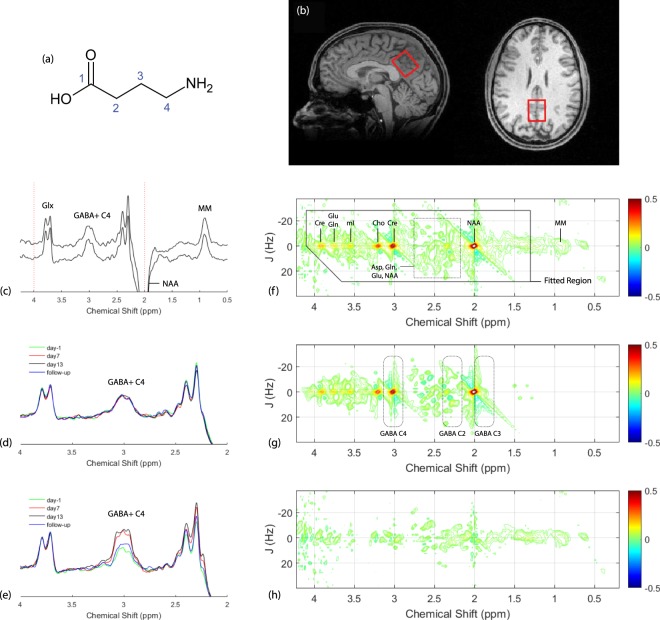
(**a**) GABA molecular structure. (**b**) Sagittal (left panel) and axial (right panel) MP-RAGE images displaying the typical positioning of the MRS voxel (red box) within the POC. (**c**) MEGA-PRESS data recorded at baseline (day -1) from a subject receiving placebo (top spectrum) and another subject receiving CPP-115 (bottom spectrum). Signal assignments are provided for GABA + C4 protons, Glx (Gln + Glu), NAA, and MM. (**d**) Expanded MEGA-PRESS spectra recorded at all four time points for a subject receiving placebo. (**e**) Expanded MEGA-PRESS spectra recorded at all four time points for a subject receiving CPP-115. (**f**) Raw 2D *J*-resolved ^1^H MRS data recorded from a single subject receiving placebo (Asp = free aspartate, mI = *myo*-inositol). (**g**) The ProFit-estimated 2D spectral fit showing the approximate 2D regions (dashed boxes) for the GABA C2, C3, and C4 proton groups. (**h**) The 2D residuum as calculated by subtracting the fit (**g**) from raw data (**f**).

Proton (^1^H) magnetic resonance spectroscopy (MRS) provides a non-invasive means for measuring a wide range of pharmacologically important low molecular weight metabolites in human brain^1^. The MRS quantification of brain γ-amino butyric acid (GABA; Fig. 1(a)), the primary inhibitory neurotransmitter in mammalian brain, has received significant interest among investigators seeking to enhance understanding of the neurochemical and neurobiological underpinnings in a range of psychiatric illnesses^2^, neurologic disease^3–6^, and substance abuse disorders^7,8^. However, at current clinical static magnetic field strengths (B_0_) of ≤3.0 Tesla (T), the use of conventional ^1^H MRS acquisition and data processing schemes is particularly unsuitable for cerebral GABA measurements for several reasons. First, GABA is present at relatively low levels throughout the human brain (~1.0 mM)^9^, and, due to low chemical shift dispersion encountered at clinical B_0_, its resonances are coincident with signals arising from high-concentration metabolite species, including creatine (Cre), glutamate (Glu), glutamine (Gln), and N-acetyl aspartate (NAA). The coincident, broad underlying ^1^H MRS component of cytosolic macromolecules (MM) presents a further challenge for GABA measures. Secondly, signal overlap is further exacerbated by scalar spin-spin (*J*)-coupling effects, which act to complicate GABA MRS resonance structures and reduce signal-to-noise ratio (SNR). Efforts to quantify GABA using standard ^1^H MRS approaches thus are generally associated unfavorable measurement precision and interscan variability^10^.

Methodological advances have improved the ^1^H MRS discrimination and quantification of brain GABA^11^. The most common approach is the *J*-editing ^1^H MRS technique and its associated variants^12,13^. In general, the *J*-editing procedure requires two separate scans where frequency-selective radiofrequency (RF) refocusing pulses (e.g. Gaussian-modulated RF waveforms) are applied in an interleaved ‘on’-‘off’ fashion to selectively refocus the GABA C4 protons at 3.0 ppm. This is achieved by applying the frequency-selective refocusing pulses either at 1.9 ppm (the ‘on’ resonance condition for the GABA C3 protons) or at 7.5 ppm (the ‘off’ resonance condition). Multiple ‘on’ resonance and ‘off’ resonance spectra are collected to build a suitable SNR, and the subsequent subtraction of ‘off’ data from ‘on’ resonance data yields a *J*-edited spectrum with a refocused GABA C4 3.0 ppm proton signal that is free of the larger dominating Cre methyl resonance. The detected 3.0 ppm signal contains co-edited contributions from homocarnosine (a dipeptide of histidine and GABA), and from MM, and the composite resonance is often termed GABA+ throughout the MRS literature. Due to its ease of implementation and minimal calibration steps prior to data acquisition, the MEGA-PRESS *J*-editing technique^12^ has found widespread use, and has become the ‘gold standard’, for investigating GABA concentration in healthy brain^14,15^ and in a variety of disease states and substance abuse disorders^3,6,8,16–19^.

A second class of ^1^H MRS metabolite-specific editing methods for isolating the GABA C4 proton resonance involves the excitation and filtration of higher-order multiple quantum coherences (MQC). These methods rely on the principle that MQCs can be stimulated for *J*-coupled proton nuclei but not for uncoupled singlet species, including the Cre methyl nuclei. For human studies, this typically has been achieved by incorporating a double-quantum filter (DQF) within single-voxel^20–24^ or multi-voxel spatial localization schemes^25^. DQF ^1^H MRS in human brain have largely been limited to methods development in healthy controls, although applications in patients with seizure disorders have been reported^26,27^. Technical details regarding MQC spin dynamics and DQF pulse sequences can be found elsewhere^28^.

Spatially localized variants of two-dimensional (2D) ^1^H MRS techniques comprise a third class of methods that have been employed for GABA quantification^29–31^. The key concept behind 2D ^1^H MRS is to encode and sample a second frequency dimension, seperating metabolite resonances over a 2D surface, which effectively enhance spectral resolution for a given B_0_. Localized 2D *J*-resolved ^1^H MRS is a commonly-employed variant that is designed to specifically encode *J*-coupling information along the second spectral dimension^31^. For GABA, 2D *J*-resolved ^1^H MRS resolves the *J*-coupled C2, C3, and C4 proton resonances along the second frequency dimension, shifting them away from the larger, overlapping, uncoupled resonances such as the Cre methyl resonance at 3.0 ppm. Historically, quantification of 2D ^1^H MRS data sets has involved integration of one-dimensional (1D) row extractions at a GABA-specific frequency position along the second dimension^29^, or calculation of volume integrals for resolved metabolite 2D ‘cross’ peaks^32,33^. Recent advances in 2D MRS fitting algorithms have significantly improved the detection and discrimination of metabolite resonances through spectral fitting of the entire 2D surface, through the incorporation of prior knowledge^34,35^. For example, the prior knowledge fitting (ProFit) algorithm reported by Schulte and Boesiger^35^ iteratively fits a series of 2D metabolite basis functions to the raw *in vivo* human data, and favorable test-rest measurement reproducibility has been demonstrated for up to nineteen brain metabolites across frontal and parietal lobe regions^35,36^. Localized 2D ^1^H MRS brain studies have been reported for both healthy brain^29,37,38^, psychiatric illness^39–41^, and substance abuse disorders^7^.

Hence, the primary advantage of localized 2D ^1^H MRS methods is the capability for simultaneously measuring all MRS-detectable metabolites in a single acquisition, including both uncoupled and *J*-coupled species. Although satisfactory test-retest reliability has been established for many metabolites, including GABA^36^, a side-by-side comparison of 2D ^1^H MRS methods with gold-standard *J*-editing ^1^H MRS techniques remains to be comprehensively evaluated. Such an assessment could be significantly helped by studies involving a GABAergic pharmacological intervention, inducing brain GABA concentration changes that can be monitored using both types of measurement techniques. Recently, we reported a study in healthy control subjects that involved administration of (1 S, 3 S)-3-amino-4-difluoromethylenyl-1-cyclopentanoic acid (CPP-115; a new-generation GABA-aminotransferase (AT) inhibitor), or placebo, for a 6, 10, or 14-day treatment period^42^. MEGA-PRESS ^1^H MRS methodologies were used to demonstrate elevated parietal-occipital cortex (POC) GABA+ levels in response to daily CPP-115 treatment, with a subsequent return to baseline levels following drug clearance. In contrast, POC GABA+ concentrations for the placebo cohort were highly-stable throughout the treatment period. In addition to MEGA-PRESS measures, we also recorded 2D *J*-resolved ^1^H MRS data from identical spatial locations. The main objective of the present report was to utilize all POC 1D (MEGA-PRESS) and 2D ^1^H MRS data obtained in our previous study^42^ to evaluate the relative performance of the two techniques for measuring GABA changes in human brain.

## Methods

### Subjects and Treatment Regimen

The University of Utah’s institutional review board (IRB) had approved the MRS protocol, and all methods were performed in accordance with the relevant guidelines and regulations required for investigations in human subjects (https://www.hhs.gov/ohrp/regulations-and-policy/belmont-report/index.html last accessed on 07/27/2018). Informed written consent was obtained from all subjects prior to study participation. Six healthy adult male subjects (mean age ± standard deviation [SD] = 34.2 ± 16.8 years) were enrolled for a double-blind, randomized, placebo-controlled study. Details regarding screening, and subject inclusion/exclusion criteria can be find in our previous report^42^. Subjects received either a single daily 80 mg dose of CPP-115 (n = 4) or placebo (n = 2) for 6, 10, or 14 continuous days, with both study drug and placebo administered as non-carbonated artificially sweetened beverages. Table 1 presents the unblinded study subject information including age, treatment (tx) allocation, and tx duration. All subjects underwent a ^1^H MRS scan at day -1 with study drug or placebo dosing initiated at day1. Two ^1^H MRS scans then were performed at day 7 and day 13, with the scans occurring 2.5 hours post-placebo or CPP-115 administration, and at approximately the same time-of-day as baseline (day -1) measures. The fourth and final ^1^H MRS measurement (follow-up) took place within the day 20 to day 23 window, to allow for a minimum 7-day washout period from the final dose day.

**Table 1:** Study subject information regarding age at the time-of-scanning, assigned treatment (Tx), and treatment duration.

Subject	Age (years)	Tx	Tx Duration (days)
1	25	CPP-115	14
2	27	Placebo	14
3	19	CPP-115	10
4	54	CPP-115	10
5	23	Placebo	6
6	57	CPP-115	6

### Data Acquisition

All ^1^H MRS measurements were performed using a 2.89 Tesla Siemens (Erlangen, Germany) Verio whole-body MRI scanner installed with VB17 Syngo software. A circularly-polarized body radiofrequency (RF) coil and manufacturer-supplied 12-channel phased array head coil were used for RF transmission and signal reception, respectively. Three-dimensional (3D) high-resolution T1-weighted, magnetization-prepared, rapid gradient echo (MP-RAGE; TR/TE/TI = 2000/3.53/1100 ms; FOV = 256 × 256 × 224 mm; 1 mm isotropic resolution) MR images were initially obtained to facilitate MRS voxel positioning, and post-hoc tissue segmentation. MEGA-PRESS and 2D *J*-resolved ^1^H MRS data were recorded from a single voxel measuring 25 × 25 × 30 mm^3^, positioned bilaterally within predominantly gray matter of the parietal-occipital cortex (POC; see Fig. 1(b)). For day-1 MRS scans, the sagittal and axial MP-RAGE slices extending through the center of the MRS voxel were saved to an image file, which subsequently was reloaded and used to aid the manual POC voxel repositioning at the three later scanning time points on a subject-specific basis. Local B_0_ shimming was performed using the manufacturer-supplied FASTMAP routine^43^, which resulted in water signal linewidths of ≤8 Hz for all voxels and subjects. MEGA-PRESS acquisition parameters were as follows: repetition time (TR) = 2000 ms, echo time (TE) = 68 ms, number of signal averages (NAV) = 256 ‘on’ and 256 ‘off’. A MEGA-PRESS spectrum also was recorded from the POC without water suppression (NAV = 16). All 2D *J*-resolved 1 H MRS measurements utilized a maximum-echo sampling scheme proposed by Schulte *et al*.^44^, using the acquisition parameters found in our previous publication^36^ (TR = 2000 ms, TE range = 31–229 ms, ΔTE = 2 ms, NAV per TE = 4). A separate 2D *J*-resolved MRS spectrum was recorded without solvent water suppression (NAV per TE = 2). Total imaging and ^1^H MRS scan measurement time did not exceed 80 minutes for each scan session.

### Data Processing

Skull stripping and whole brain tissue-type segmentation was performed on MP-RAGE images using the Brain Extraction Tool (BET)^45^ and Fast Automated Segmentation Tool (FAST)^46^, respectively, which are provided with the freely-available FMRIB software library^47^. MATLAB (The MathWorks, Natick, MA) functions then were used to extract the 3D volume corresponding to the positioned MRS voxel to obtain within-voxel gray matter (GM), white matter (WM) and cerebrospinal fluid (CSF) tissue content for each subject. The GM and WM fractional content was calculated as a percentage of total brain tissue, e.g. 100 × GM ÷ (GM + WM).

GABA-edited MEGA-PRESS ^1^H MRS data were processed and quantified using the freely-available Gannet software^48^ using the raw Siemens ‘TWIX’ data format. The software’s default preprocessing steps, peak fitting parameters, and subtraction procedures were used for all MEGA-PRESS data. To extract POC GABA+ concentration, a total creatine (Cre) concentration of 8 mM was assumed and applied as a multiplication factor to the GABA+: Cre ratio. The MEGA-PRESS off-resonance data also was used to generate a regular 1D PRESS spectrum for each subject, which were analyzed using the Linear Combination (LC)-Model software^49^ employing a simulated basis set created for B_0_ = 2.89 T and TE = 68 ms. LC-Model outputted metabolite:Cre ratios were analyzed for several compounds including NAA, Cho, Gln, Glu, mI, and GABA. The ProFit algorithm^35^ was used to reconstruct and subsequently fit all POC 2D *J*-resolved MRS data as detailed elsewhere^36^. An important post-processing step performed by ProFit is the required row-dependent phase-shift that reduces the bandwidth along the second dimension to ±125 Hz. ProFit-estimated POC metabolite levels, including GABA, were expressed as the default GABA:Cre ratio. The relative stability of the Cre denominator was assessed for both measurement techniques by normalizing its signal amplitude with that of a short TE unsuppressed water signal (TE = 31 ms, NAV = 2) acquired from the relevant voxel location. The unsuppressed water signal also had been corrected for within-voxel CSF content as described elsewhere^36^.

### Data Analysis

Statistical analysis, including analysis of variance with repeated measures (ANOVA-RM), was performed using MATLAB and OriginPro (2016, Northampton, MA). Coefficient of variation (CV) was expressed as 100 × SD ÷ data mean. To quantify the POC GABA changes induced by CPP-115 vs placebo, the effect size was calculated for the day 7 and day 13 time points for both measurement techniques. Subject data from both cohorts were included to calculate effect sizes using the relevant pooled SD.

Correlation analysis (Pearson’s rho, *r*) and Bland-Altman (BA) analysis were performed after converting day 7, day 13, and follow-up GABA+ concentration (MEGA-PRESS) and GABA:Cre ratios (2D *J*-resolved) to the % of the respective baseline value (day-1). Whereas correlation analysis explores the relationship between two techniques, BA analysis enables a quantification of agreement between the two measurements by studying mean differences and generating limits of agreement. The BA plot was constructed by plotting the difference in % baseline between the two measurement techniques, against the mean % baseline of the two measurement techniques. Additional BA outputs included calculation of the coefficient of repeatability (RPC), which was performed after ensuring the within-subject differences met the Shapiro-Wilk test for normality. The RPC was calculated as 1.96 × standard deviation (SD) of the within-subject data differences^50^.

The Gannet GABA+ and ProFit GABA fit estimates were interrogated using the outputted GABA+ fit error and Cramer-Rao Lower Bound (CRLB) values, respectively. The Gannet GABA+ fit error is calculated as a percentage of the SD of the residual (raw data minus fit) divided by GABA+ peak amplitude^48^. Calculation of the ProFit CRLB has been described elsewhere^35^.

## Results

Table 2 presents mean within-voxel tissue data for both treatment groups. The CV values presented were obtained by first determining the CV for each subject, and then calculating the mean within-subject CV for each cohort.

**Table 2:** Tissue (GM and WM) tissue fractions calculated for both treatment groups.

Cohort	GM	CV (GM)	WM	CV (WM)
CPP-115	61 ± 6	1 ± 1	39 ± 6	4 ± 2
Placebo	62 ± 5	1 ± 1	38 ± 5	3 ± 2

The mean within-subject coefficient of variation (CV) also is presented for both tissue types. All values are expressed as mean % values ± SD.

**Figure 2:**
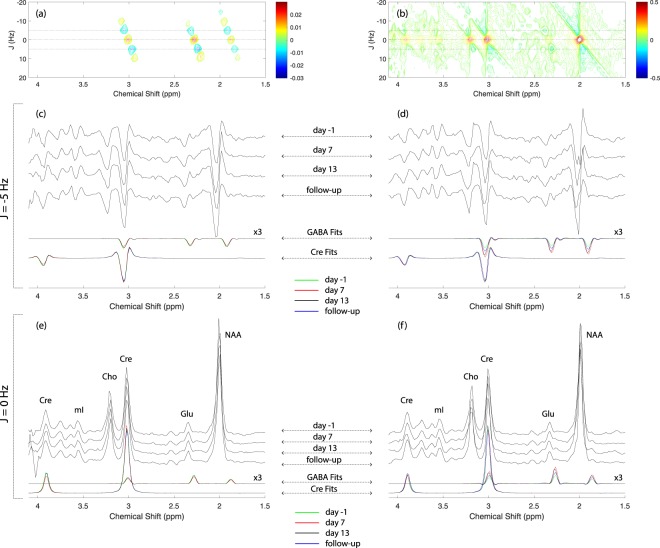
(**a**) Simulated 2D *J*-resolved ^1^H MRS spectrum of GABA showing its C2, C3, and C4 resonances centered at 2.28, 1.9, and 3.0 ppm, respectively. The spectrum is presented in phase-sensitive mode (real component) and signal phases can be appreciated using the colorbar provided. For the GABA resonances, maximum positive signals reside along *J* = 0 Hz whereas, for components *J* ≠ 0 Hz components, maximum negative GABA signals are detected at approximately *J* = ±5 Hz. The horizontal black lines depict the *J* = 0 Hz and *J* = ±5 Hz rows. (**b**) Representative i*n vivo* 2D *J*-resolved 1 H MRS spectrum recorded from Subject 2 (day -1) for direct comparison with the simulated GABA data. The simulated and *in vivo* data are plotted with different arbitrary 2D scaling factors for visualization purposes. (**c**,**d**) then display the *J* = −5 Hz row extractions, for all four MRS time points, in subjects receiving placebo (Subject 2) and CPP-115 (Subject 1), respectively. The ProFit-estimated GABA and Cre spectral fits along *J* = −5 Hz also are displayed for each MRS visit (refer to color coding). Panels (e,f) show the corresponding *in vivo* time course MRS data and the resulting GABA and Cre ProFit-estimated spectral fits along *J* = 0 Hz for Subject 2 (placebo) and Subject 1 (CPP-115), respectively. Signal assignments are provided for major resonances in (**e**,**f**). Identical 1D scaling factors were used to present (**c**,**d**), whereas a different yet identical scaling factor was used to present (**e**,**f**). For panels (c) through (**e**), the 1D scaling used to present the ProFit-estimated GABA fits was enhanced threefold (x3) relative to the corresponding Cre fits.

**Figure 3:**
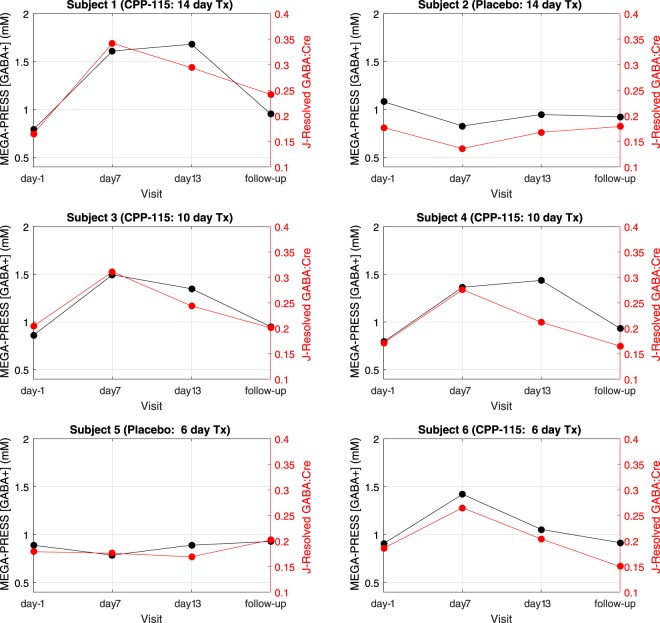
Time course data for all six participants showing the change in POC GABA levels across the four scan sessions, measured using MEGA-PRESS (black data) or 2D *J*-resolved ^1^H MRS (red data). Each of the six plots are identified by subject number, with the tx allocation and tx duration also provided.

Baseline (day-1) MEGA-PRESS data recorded from a subject receiving placebo (subject 2) and a subject receiving CPP-115 (subject 1) are displayed in Fig. 1(c). The chemical shift expanded spectra (0.5–4.25 ppm) shown in Fig. 1(d,e) show the MEGA-PRESS data recorded at all four scanning time points for subject 2 (placebo) and subject 1 (CPP-115), respectively. Figure 1(f–h) show the raw baseline 2D *J*-resolved ^1^H MRS data (top panel), the ProFit spectral fit (middle panel), and the residual data (raw data minus fit; bottom panel) recorded from subject 2. Further visualization of the 2D *J*-resolved MRS data is presented in Fig. 2, which first shows a simulated 2D GABA basis spectrum (a) as utilized by the ProFit software, and a representative *in vivo* dataset (b) for direct comparison. Both 2D MRS spectra are presented in the phase-sensitive mode (real component) and analysis of the basis spectrum in Fig. 2(a) shows maximum negative GABA peaks residing at approximately *J* = ±5 Hz, and maximum positive signals through *J* = 0 Hz. For two study subjects receiving either placebo or CPP-115, 1D row extractions taken through *J* = ±5 Hz and *J* = 0 Hz are displayed in Fig. 2(c–f), showing the raw *in vivo* data at each of the four individual time points. Also displayed in Fig. 2(c–f) are the corresponding 1D *J* = −5 Hz and *J = *0 Hz row extractions taken directly from the Cre and GABA 2D spectral fits estimated by the ProFit software (additional details are provided in the Fig. 2 legend). Figure 3 displays, for all six subjects, the quantitative POC GABA changes as derived using the MEGA-PRESS and 2D *J*-resolved ^1^H MRS methodologies. For the placebo group (n = 2), mean GABA CV values of 9% and 10% were calculated for the MEGA-PRESS and 2D *J*-resolved MRS methods, respectively. For the CPP-115 group (n = 4), mean GABA CV values of 33% and 25% were calculated for the MEGA-PRESS and 2D *J*-resolved MRS methods, respectively. Table 3 displays the CV values for Cre reference stability (based on water normalization) for both treatment groups and measurement techniques. Also displayed in Table 3 are the day 7 and day 13 effect sizes calculated for GABA changes for the CPP-115 treatment group.

**Table 3:** Mean within-subject Cre CV values presented as % ± SD values for both measurement techniques and treatment cohorts.

Cohort	Technique	CV (Cre:water)	ΔGABA Effect Size (day 7, day 13)
CPP-115	MEGA-PRESS	4 ± 1	7.2, 2.1
	2D *J*-Resolved	7 ± 2	4.3, 2.0
Placebo	MEGA-PRESS	5 ± 2	N/A
	2D *J*-Resolved	9 ± 4	N/A

For both techniques, the final column shows the effect size for drug-induced GABA changes at day 7 and day 13. Note that the Cre CV and GABA effect size metrics are based on CSF-corrected unsuppressed water and Cre normalization, respectively (see text for additional details).

**Figure 4:**
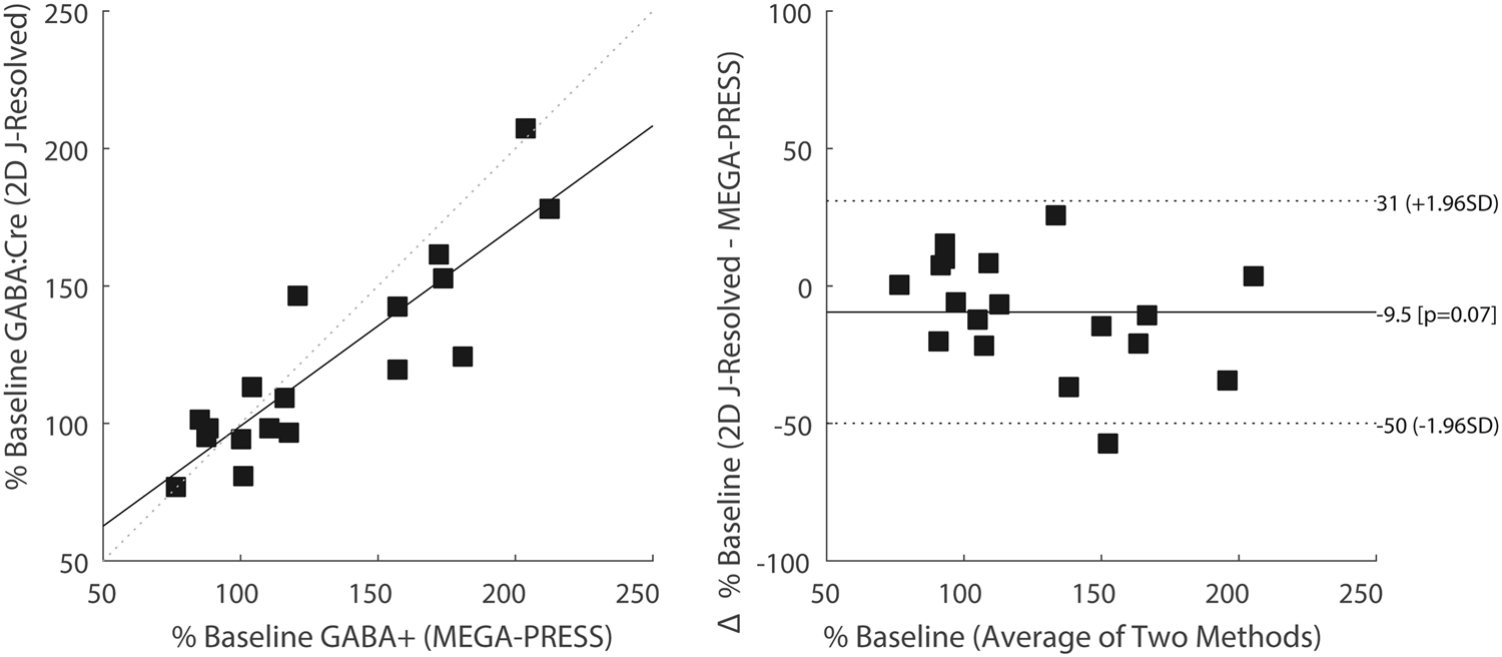
(**a**) Plot of % baseline GABA:Cre measures for 2D *J*-resolved ^1^H MRS versus % baseline GABA+ measures for MEGA-PRESS. The dashed line represents the identity line (y = x). (**b**) The constructed BA plot, displaying the difference in % baseline GABA for both techniques, versus the average % baseline GABA for the two methods. The solid horizontal line denotes the mean difference (−9.5%), and the dotted lines represent the ± RPC values (i.e. ±1.96 × SD).

Figure 4(a) plots % baseline GABA:Cre for 2D *J*-resolved MRS versus % baseline GABA+ for MEGAPRESS (n = 18, *r*^2^ = 0.77, p < 0.001). The corresponding BA analysis for the two techniques displayed in Fig. 4(b) and, at the 5% level, normality could not be rejected for the within-subject % GABA differences (p = 0.9). BA analysis using all data points showed a mean offset of ~10% with a RPC value of ~40%. Based on the time courses shown in Fig. 3, removal of the potential outlier (subject 4, day 13) and re-running BA analysis reduced the mean offset deficit to ~6% (RPC reduced to 34%). To effectively use data points deemed free from the effects of CPP-115, BA analysis also was ran using all placebo non-baseline data points and only the follow-up data points for CPP-115 (data not shown). Although a weaker correlation was observed (n = 10; *r*^2^ = 0.4), the BA mean offset was almost zero (0.8%; RPC = 30).

**Figure 5:**
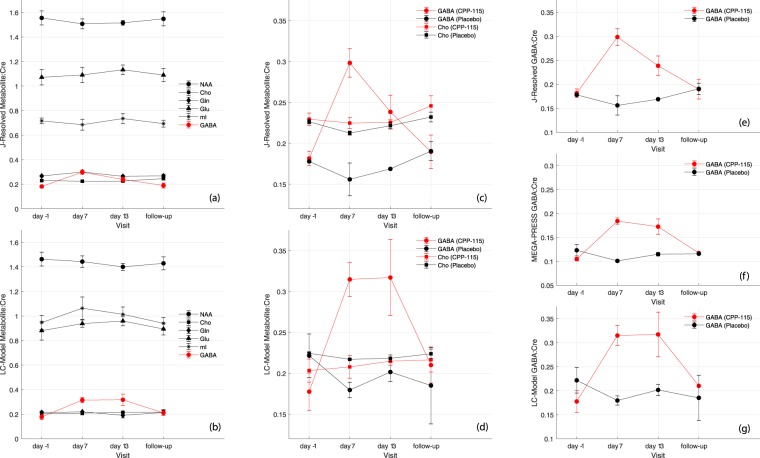
Mean (±standard error [SE]) metabolite:Cre level time course data obtained for the CPP-115 cohort (N = 4) using (**a**) 2D *J*-resolved ^1^H MRS and ProFit and (**b**) 1D ^1^H MRS (TE = 68 ms) and LC-Model. (**c**,**d**) Show expanded zoom plots of (**a**,**b**) to show GABA:Cre time course data presented for both cohorts together with the corresponding Cho data. Calculated mean (±SE) GABA:Cre time course data for both treatment groups for (**e**) 2D ^1^H MRS and ProFit, (**f**) MEGA-PRESS ^1^H MRS and Gannet, and (**c**) 1D ^1^H MRS and LC-Model.

For the CPP-115 cohort, Fig. 5(a,b) show the Cre-normalized time course data of six selected metabolites measured using the 2D ^1^H MRS/ProFit and 1D ^1^H MRS/LC-Model methods, respectively. Figure 5(c,d) show expansions of (a) and (b), but overlays Cre-normalized POC GABA with POC Cho data for both treatment groups. For 2D ^1^H MRS and ProFit, ANOVA-RM analysis revealed highly significant changes for POC GABA levels in the CPP-115 cohort (*F*_3,9_ = 20.5, p < 0.001), but not for placebo (*F*_3,3_ = 1.5, p = 0.4). For both groups, ANOVA-RM analysis revealed negligible changes for the other five metabolites. Figure 5(e–g) show the mean GABA:Cre time course data for both treatment groups, and for each of the three data acquisition and analysis approaches.

**Figure 6:**
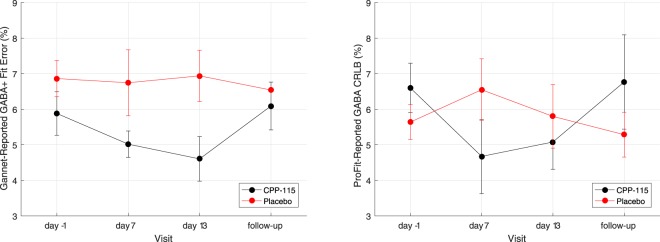
(**a**) The mean (±SE) Gannet-reported GABA fit error values presented for both cohorts and across all MRS scanning time points. (**b**) The mean ProFit-reported GABA CRLB (±SE) values shown for both cohorts across all MRS scanning time points. See text for statistical details.

Spectral fitting estimates derived for GABA using the MEGA-PRESS/Gannet and 2D *J*-resolved MRS/ProFit methods are presented for all time points in Fig. 6. For MEGA-PRESS, the ANOVA-RM showed a trend towards significance for the CPP-115 group (*F*_3,9_ = 3.59, *p* = 0.06) but not for the placebo cohort (*F*_3,3_ = 0.21, *p* = 0.90). For 2D *J*-resolved ^1^H MRS, the ANOVA-RM was statistically significant for the CPP-115 group (*F*_3,9_ = 3.91, *p* = 0.05), but not for the placebo cohort (*F*_3,3_ = 0.45, *p* = 0.74). The mean ProFit-reported CRLB values for GABA were 6 ± 2% and 6 ± 1% for the CPP-115 and placebo cohorts, respectively. The corresponding LC-Model CRLB values for the 1D ^1^H MRS data were 15 ± 5% and 16 ± 2%.

## Discussion

Improved ^1^H MRS data acquisition and processing methods have significantly advanced the quality and reproducibility of brain GABA measures in human brain^48,51,52^. Future studies will be critical for understanding GABA abnormalities in studies of neuropsychiatric and neurological disease states, as well as for monitoring the efficacy of pharmacological therapeutics^53–55^ and medical device interventions^56^. The *J*-editing approach remains the gold-standard for *in vivo* GABA measures across most research institutions, and, owing to its relatively straightforward implementation, the MEGA-PRESS pulse sequence is the most commonly-employed variant for GABA *J*-editing. In contrast, the utilization of 2D ^1^H MRS methods for brain GABA measures is less common. Issues that have prevented a more widespread use of 2D ^1^H MRS for biological applications include the lengthier associated measurement times, and access to robust 2D MRS fitting algorithms. Efforts to reduce total 2D MRS measurement time have recently found traction through the introduction of non-uniform weighted sampling (NUWS) procedures^57–60^, and quantitative treatment of 2D ^1^H MRS data has been advanced through automated methods such as ProFit^35^. Highly favorable inter- and intra-subject reproducibility 2D ^1^H MRS and ProFit measures for cerebral GABA and a wide range of other neurometabolites including free aspartate (Asp), Cho, Cre, Gln, Glu, glutathione, glycine, mI, and NAA have been reported^36^. Promising test-retest metrics aside, several important performance-related questions remain. First, how effective are 2D ^1^H MRS methods and state-of-the-art 2D fitting algorithms for monitoring changes in low concentration metabolite species, such as GABA, following pharmacotherapy or medical device treatment, and how do the detected GABA concentration changes compare against the current gold-standard methodology? Second, can the induced changes, detected using 2D ^1^H MRS methods, be shown to be specific to the metabolite under investigation? The present study aimed to address these questions by comparing POC *J*-edited (MEGA-PRESS) and 2D *J*-resolved ^1^H MRS data obtained from healthy individuals receiving chronic doses of CPP-115 or placebo. Using this unique dataset, we were able to evaluate the performance of each method, and a comparison of drug-induced GABA concentration changes as detected using MEGA-PRESS and 2D ^1^H MRS is qualitatively and quantitatively discussed hereafter.

Importantly, CPP-115 does not induce detectable macroscopic POC tissue changes, as stable within-voxel GM, WM, and CSF content was detected for both tx groups. MEGA-PRESS ^1^H MRS spectra recorded from individuals receiving CPP-115 clearly showed increases in POC GABA that returned to baseline following washout. MEGA-PRESS ^1^H MRS spectra obtained from the placebo cohort showed stable GABA peak amplitudes at all four MRS scanning time points. To demonstrate possible GABA changes via 2D ^1^H MRS we selected to display row extractions at specific frequencies along the second (*J*) dimension of raw *in vivo* data and the corresponding ProFit-estimated spectral fits. We analyzed the 1D dimensional rows taken through *J* = −5 Hz and *J* = 0 Hz, as inspection of the phase-sensitive 2D *J*-resolved ^1^H MRS basis spectrum for GABA revealed maximum negative and maximum positive metabolite peaks along these frequencies. This is contradictory to previous reports that have used 1D row extractions through *J* ~ ±7 Hz, which is the approximate *J*-coupling constant associated with the GABA C4 proton resonance^7,29^. Those investigations, however, typically involve analysis of 2D ^1^H MRS data presented in magnitude-mode, and it should be noted that calculation and presentation of the GABA 2D *J*-resolved ^1^H MRS basis spectrum acts to restore the GABA C4 proton cross peaks positioned at *J* ~ ±7 Hz (data not presented). Row *J* = 0 Hz data, on the other hand, is mathematically equivalent to directly averaging across all TE steps (TE-averaged ^1^H MRS), which simplifies the ^1^H MRS baseline and metabolite peak structures and has found utility Glu detection^61^. For the placebo condition, *J* = −5 Hz and *J* = 0 Hz analysis showed highly-stable ProFit-estimated spectral fits for both Cre and GABA, whereas for the CPP-115, treatment group, the same analysis revealed increased GABA levels at days 7 and 13, and a return towards baseline at follow-up. The drug-induced GABA modulations detected for the CPP-115 condition are accompanied by stable Cre fits along *J* = −5 Hz and *J* = 0 Hz. The reader is encouraged to recognize that ProFit does not treat the *in vivo* 2D 1 H MRS data on a row-by-row basis, but instead applies a linear combination of specified 2D metabolite basis functions to fit the entire 2D spectral surface^35^.

The individual time course data showed that GABA changes measured using MEGA-PRESS were generally well-mirrored by the corresponding levels obtained using 2D *J*-resolved ^1^H MRS. For the CPP-115 cohort, elevated MEGA-PRESS GABA levels were followed by similar changes in 2D *J*-resolved GABA levels, and only subject 4 showed a substantial difference for the two techniques at the day 13 measure. Elevated GABA levels returned to baseline values for both measurement techniques, demonstrating that the two methods are comparable for tracking (i) GABA-AT inactivation, and (ii) protein resynthesis following washout. For the placebo cohort, closely similar GABA CV values were observed for both measurement techniques (9–10%), whereas, due to the increased GABA concentrations at day 7 and day 13, significantly higher GABA CV values were calculated for the CPP-115 tx group. These changes were based on Cre-normalization, therefore it was encouraging to note favorable denominator stability (based on tissue water) for both measurement techniques (CV < 10%).

Prior to quantitative correlation and BA analysis, the units for GABA measures for both measurement techniques were standardized by considering the day 7, day 13, and follow-up GABA measures as % baseline (day -1) values. Although a significant linear relationship was observed for the two techniques (*r*^2^ ~ 0.8), a clearer understanding of their agreement can be appreciated through BA analysis, which indicates that the MEGA-PRESS method detects ~10% more GABA signal change, compared to baseline, relative to the 2D *J*-resolved MRS approach. This appears to be somewhat driven by mean % baseline GABA levels of greater than 100% baseline, which corresponds to CPP-115 measures at days 7 and 13. Overall, the constructed limits of agreement (RPC) from BA analysis infer that % baseline GABA levels obtained using 2D *J*-resolved ^1^H *J*-resolved MRS and ProFit may be no greater than 50% below or 31% above MEGA-PRESS measures. The BA data also revealed encouraging agreement between the two measurement techniques (i.e. an almost zero mean offset and RPC = 30%) when including data points that were judged free from the effects of CPP-115 administration.

The larger GABA signal change associated with MEGA-PRESS BA analysis also is reflected by the larger effect size calculated its day 7 measures. If MEGA-PRESS is considered as the reference method, the reasoning behind reduced estimates (i.e. lower effect size) for 2D *J*-resolved MRS measures at higher GABA concentrations remains unclear. One possible intriguing explanation is conversion of GABA to homocarnosine, a known neuromodulator and storage compound for GABA, and how that metabolism relates to the fitting approaches used by the Gannet and ProFit software. Our previous report showed (i) significant increases in homocarnosine levels in response to CPP-115 administration, which were estimated to be the same order of magnitude if not greater than detected GABA elevations, and (ii) negligible change in MM content associated with CPP-115 GABA-AT inhibition^55^. Whereas the Gannet software fits a single Gaussian line shape to the edited composite 3.0 ppm peak in MEGA-PRESS data, the ProFit algorithm applies a 2D basis function for GABA that constrains all chemical shift and *J*-coupling information for each of its methylene proton groups (as well as tailored 2D basis spectra for other metabolites). It is thus possible that the GABA changes detected using 2D *J*-resolved MRS and ProFit are reflective of GABA modulation at later time points, i.e. after a chronic period of CPP-115 dosing, carnosine synthase controls GABA levels with its conversion to homocarnosine. This might be characterized by a rise of GABA concentration at day 7, with subsequent decreases at day 13 following chronic exposure to CPP-115. Indeed, three of the subjects receiving CPP-115 for >10 days showed 2D *J*-resolved ^1^H MRS GABA concentration time courses that would fit this model. Other biological mechanisms and sources of method-specific error could play their part in the differences observed between the GABA measurement techniques, yet the present dataset and similar types of BA analysis could prove useful for testing alternative time-domain (e.g. jMRUI^62^) and frequency-domain (e.g. LC-Model^49^) approaches for quantifying MEGA-PRESS GABA-edited data, as well as the development of novel 2D MRS quantification algorithms.

To establish whether 2D ^1^H MRS detection of drug-induced changes were specific to GABA, we analyzed the metabolite:Cre time courses for multiple POC compounds, including Cho, GABA, Gln, Glu, mI, and NAA. Highly-stable time courses were observed for all other metabolites suggesting that CPP-115 has negligible effect on certain measures of neuronal function (stable NAA), cell membrane turnover/synthesis (stable Cho), glial activation (stable mI), and glutamatergic metabolism (stable Gln and Glu). The elevated GABA levels are evident when directly compared with the Cho time course from both tx groups, as well as the GABA time course from the placebo group. These observations are reinforced by the drug-induced GABA modulation and relative stability of Cho, Gln, Glu, mI, and NAA levels obtained through LC-Model analysis of the off-resonance 1D ^1^H MRS MEGA-PRESS data. However, the increased variance and diminished fit precision (i.e. significantly higher CRLBs) associated with 1D ^1^H MRS and LC-Model analysis should be noted. Increased GABA concentration also is mirrored by changes in the fitting error observed for the 2D ^1^H MRS and MEGA-PRESS techniques. For MEGA-PRESS a trend towards significantly reduced GABA fitting errors was detected for the CPP-115 cohort but not for the placebo group, whereas for 2D *J*-resolved ^1^H MRS and ProFit, the GABA CRLB values for the CPP-115 group reached significance at the *p* = 0.05 level, but not for placebo. The Gannet-reported fitting errors and ProFit-reported CRLB calculations are inversely-driven by SNR, and their values are expected to decrease with drug-induced increases in GABA concentration^35,48^.

A potential limitation of this methods comparison concerns the relative sensitivity of the two measurement techniques. From a fundamental SNR perspective, MEGA-PRESS methodology should afford approximately a 13% higher SNR than the 2D *J*-resolved ^1^H MRS approach, i.e. equivalent repetition times, 512 versus 400 total signal averages for MEGA-PRESS and 2D ^1^H MRS, respectively. However, owing to the different sampling schemes used for MEGA-PRESS (single TE, half-echo sampling) and 2D ^1^H MRS (multiple TEs, maximum-echo sampling), a better appreciation of relative sensitivity is accomplished using the analytical derivations provided by Schulte *et al*.^44^ that require knowledge of *in vivo* metabolite spin-spin (T_2_) relaxation times, the observed T_2_ (T_2_*), and the total sampling time along the second dimension (TS1) for 2D *J*-resolved ^1^H MRS. Assuming a GABA T_2_ relaxation of 88 ms^63^, the measured T_2_* from the current dataset (53 ms)^55^, and a TS1 of 200 ms, it can be shown that the sensitivity of the maximum-echo sampled 2D *J*-resolved ^1^H MRS measures is approximately 55% that of a half-echo sampled short-TE PRESS acquisition. This analysis assumes comparison of 2D *J*-resolved ^1^H MRS with a PRESS acquisition employing the shortest TE within its sampling range (TE = 31 ms), and the relative sensitivity of the 2D ^1^H MRS methods utilized here is expected to be higher when considering the TE associated with MEGA-PRESS (TE = 68 ms). This analysis also assumes identical experimental durations, so the sensitivity gains may be offset due to SNR arguments introduced earlier. Nevertheless, for *in vivo* GABA measures, we approximate a relative sensitivity of 50% for the maximum-echo sampled 2D *J*-resolved ^1^H MRS approach versus MEGA-PRESS methods. Pulse sequences, GABA proton spin response, and resulting signal yield then are compared for the two measurement techniques. For 2D *J*-resolved ^1^H MRS, all GABA proton signals are sampled and retained in the final 2D spectrum, whereas ~50% of the GABA C4 proton signal at 3.0 ppm is retained for analysis in reconstructed MEGA-PRESS data (only the outer wings of the GABA C4 proton multiplet are constructively averaged following data subtraction). For the present study we thus expect a comparable relative technique sensitivity, although spectral simulation studies designed to rigorously assess these concepts are warranted.

These findings demonstrate the direct comparison between spatially localized 2D ^1^H MRS techniques and *J*-editing ^1^H MRS methods for GABA quantification in human brain. Technique comparability was greatly facilitated through administration of a GABA-AT inhibitor, which induced robust modulation of intracellular GABA concentration and permitted subsequent qualitative and quantitative time course evaluation. In general, data from the 2D ^1^H MRS acquisitions closely-mirrored *J*-editing and, for GABA, it is expected that the methods can be substantially improved with further methodological improvements including MM and homocarnosine analysis/fitting, and integration of realistic basis spectra (i.e. incorporating the effects of spatial localization). The performance of 2D 1H *J*-resolved MRS for measuring a range of other important neurometabolites with *J*-coupled spin systems, including Gln and Glu, is expected be at least comparable with GABA measures given their larger intracellular concentration. The integration of data acquisition schemes based on NUWS methods^57–60^, together with integration of multi-voxel sampling methods^64–66^, are continuing to improve temporal resolution of localized 2D ^1^H MRS measurements for human applications. Considering those developments, together with the data presented here, the future potential for simultaneous whole-brain mapping of multiple metabolites, including GABA, with 2D ^1^H MRS on clinical MRI/MRS systems is particularly encouraging.

## Data Availability

The anonymized datasets generated during and/or analyzed during the current study are available from the corresponding author upon request.
